# *Bactris gasipaes* Kunth var. *gasipaes* complete plastome and phylogenetic analysis

**DOI:** 10.1080/23802359.2022.2109437

**Published:** 2022-08-26

**Authors:** Maria Camila Buitrago Acosta, Rommel Montúfar, Romain Guyot, Cedric Mariac, Timothy J. Tranbarger, Silvia Restrepo, Thomas L. P. Couvreur

**Affiliations:** a Laboratorio de Micología y Fitopatología, Universidad de los Andes, Bogotá, Colombia; b Facultad de Ciencias Exactas y Naturales, Pontificia Universidad Católica del Ecuador, Quito, Ecuador; c DIADE, Univ Montpellier, CIRAD, IRD, Montpellier, France; d Department of Electronics and Automation, Universidad Autónoma de Manizales, Manizales, Colombia

**Keywords:** Chloroplast, plastome, Novoplasty, peach palm, phylogeny

## Abstract

*Bactris gasipaes* var. *gasipaes* (Arecaceae, Palmae) is an economically and socially important plant species for populations across tropical South and Central America. It has been domesticated from its wild variety, *B. gasipaes* var. *chichagui*, since pre-Columbian times. In this study, we sequenced the plastome of the cultivated variety, *B. gasipaes* Kunth var. *gasipaes* and compared it with the published plastome of the wild variety. The chloroplast sequence obtained was 156,580 bp. The cultivated chloroplast sequence was conserved compared to the wild type sequence with 99.8% of nucleotide identity. We did, however, identify multiple Single Nucleotide Variants (SNVs), insertions, microsatellites and a resolved region of missing nucleotides. A SNV in one of the core barcode markers (*matK*) was detected between the wild and cultivated accessions. Phylogenetic analysis was carried out across the Arecaceae family and compared to previous reports, resulting in an identical topology. This study is a step forward in understanding the genome evolution of this species.

## Introduction

The palm family Arecaceae (Palmae) consists of more than 2,500 species (Dransfield et al. 2008), including macroeconomical important taxa. The only fully-domesticated palm from the Neotropics since pre-Columbian times is *Bactris gasipaes* Kunth (Clement 1988). This species is cultivated from Brazil to Mexico, where it is important for local populations and a staple food for Ameridian people (Clement 1988; Graefe et al. 2013). Two varieties are recognized within the species: the cultivated or domesticated variety *B. gasipaes* var. *gasipaes* and the wild variety *B. gasipaes* var. *chichagui* (Henderson 2000; Couvreur et al. 2007). Both varieties are quite similar in their overall vegetative morphology. However, the fruits from the domesticated type are much larger (3–8 cm in diameter versus 1–2 cm in the wild type, Henderson 2000) with a thicker mesocarp, being up to two hundred times heavier than the wild fruit, which represents a clear domestication syndrome (Clement et al. 2021). The fruits of *Bactris gasipaes* have traditionally been consumed as a source of carbohydrates and lipids throughout the Neotropics, and are generally prepared as fermented drinks (e.g. chicha), flours or eaten as such after cooking (Clement and Urpí 1897). The more recent and modern commercial exploitation of *B. gasipaes* palm hearts is widely extended into tropical lowlands of Central and South America, as well as the use of its wood for furniture and construction (Montúfar and Rosas 2013, Couvreur et al. 2006).

Molecular studies have focused on characterizing its local diversity and germplasm collections, as well as exploring the origins of the domestication process using molecular markers (SSRs, RAPD) and chloroplastic sequences (Hernández-Ugalde et al. 2011; Rodrigues et al. 2005; Galluzzi et al. 2015; Clement et al. 2017; Santos da Silva et al. 2021) or to understand the genetic relationships and gene flow between both varieties (Couvreur et al. 2006, Couvreur et al. 2007; Hernández-Ugalde et al. 2011). In this context, it is necessary to develop new genomic tools to explore evolutionary, ecological and agricultural issues, in particular to better unravel its intriguing domestication history across the Neotropics (Galluzzi et al. 2015; Clement et al. 2017). A complete chloroplast sequence for *B. gasipaes* Kunth var. *chichagui* (the wild variety) was recently published (Santos da Silva et al. 2021) and opened the way to explore the origins of its domestication. The goal of this work was (i) to characterize the complete plastome of *B. gasipaes* var. *gasipaes* (domesticated variety), (ii) compare it with the plastome of *B. gasipaes* var. *chichagui* (wild variety), and (iii) reconstruct a phylogenetic tree using this newly acquired plastome with different species of the Arecaceae family.

## Materials and methods

We sampled a domesticated individual of *Bactris gasipaes* Kunth var. *gasipaes* from North-Western Ecuador, in the Maship area (farm of Alejandro Solano, 0°10′54.1″N 78°54′37.1″W). The fruits of this specimen were also collected but were immature at the time, and thus no measurements were made. The young palm heart was collected in the field and immediately conserved in liquid nitrogen until total DNA was extracted the protocol of Mariac et al. (1970). The NGS library preparation follows Mariac et al. (2014). Total DNA extracted from leaves was sequenced (paired end, 150 bp) using Novaseq 6000 Illumina platform at the Novogene Co., Ltd. facilities. Sequence data were submitted to NCBI SRA section under the BioSample accession SAMN27503645.

Reads obtained were filtered by quality using Fastp. Kraken2 was used to filter possible contamination in the reads from other organisms using a database (PlusPFP) (Wood et al. 2019). NOVOPLASTY (Dierckxsens et al. 2017) was used to assemble the *Bactris gasipaes* var. *gasipaes* chloroplast sequence based on the *Elaeis guineensis* chloroplast reference genome (NC_017602.1). Ten million pair-end reads were sampled and used.

CPGAVAS2 (Shi et al. 2019) was used to annotate the chloroplast sequence, graphical representation was obtained using Chloroplot (Zheng et al. 2020). A dot-plot was constructed to compare *B. gasipaes* var. *gasipaes* and *B. gasipaes* var. *chichagui* chloroplast sequences using Gepard (Krumsiek et al. 2007). A pairwise alignment using BLASTn and diffseq (EMBOSS) was constructed with *B. gasipaes* Kunth var. *chichagui,* to analyze the presence of Single Nucleotide Variants (SNVs), insertions and deletions (Altschul et al. 1990; Aggeli et al. 2018). IRscope was used to analyze chloroplast junctions between inverted repeats and single copy regions (Amiryousefi et al. 2018). Finally, a phylogenetic tree was constructed between closed species of the family Arecaceae.

We sampled 17 outgroup palm species covering all subfamilies (Baker et al. 2009), and one species from the sister family to palms Dasypogonaceae (*Dasypogon bromeliifolius*) (Givnish et al. 2018). We also included the recently sequenced plastome of the wild variety *B. gasipaes* var. *chichagui*. No large rearrangement was identified between the sequences using dot-plot alignments. Plastomes were aligned using MAFFT version 7 (Katoh et al. 2019). Phylogenetic inferences were carried out using RAxML version 7.2.7 using GTRCAT substitution model with all sites of the chloroplast sequence without gaps using the maximum likelihood method with bootstrap of 1,000 replicates (Stamatakis 2015).

The botanically vouchered specimen was deposited at the Herbario QCA (https://bioweb.puce.edu.ec/QCA; Pontificia Universidad Católica del Ecuador, Quito; thomas.couvreur@ird.fr) and WAG (Naturalis, Leiden, The Netherlands) herbaria under the number *Couvreur & Tranbarger 1192* (collected 28 august 2018) and the DNA is deposited at the IRD center Montpellier, France (http://www.ird.fr; UMR DIADE; thomas.couvreur@ird.fr)

## Results and discussion

This work exploits the ability of NGS sequencing to produce a large quantity of reads in a very short time from total DNA. These reads can be used to explore genome composition, identify variations and SNP markers, or assemble chloroplast genomes. Here, we obtained the chloroplast sequence of the domesticated variety of *B. gasipaes*. This is an important step toward understanding the evolution of this species.

**Figure 1.**
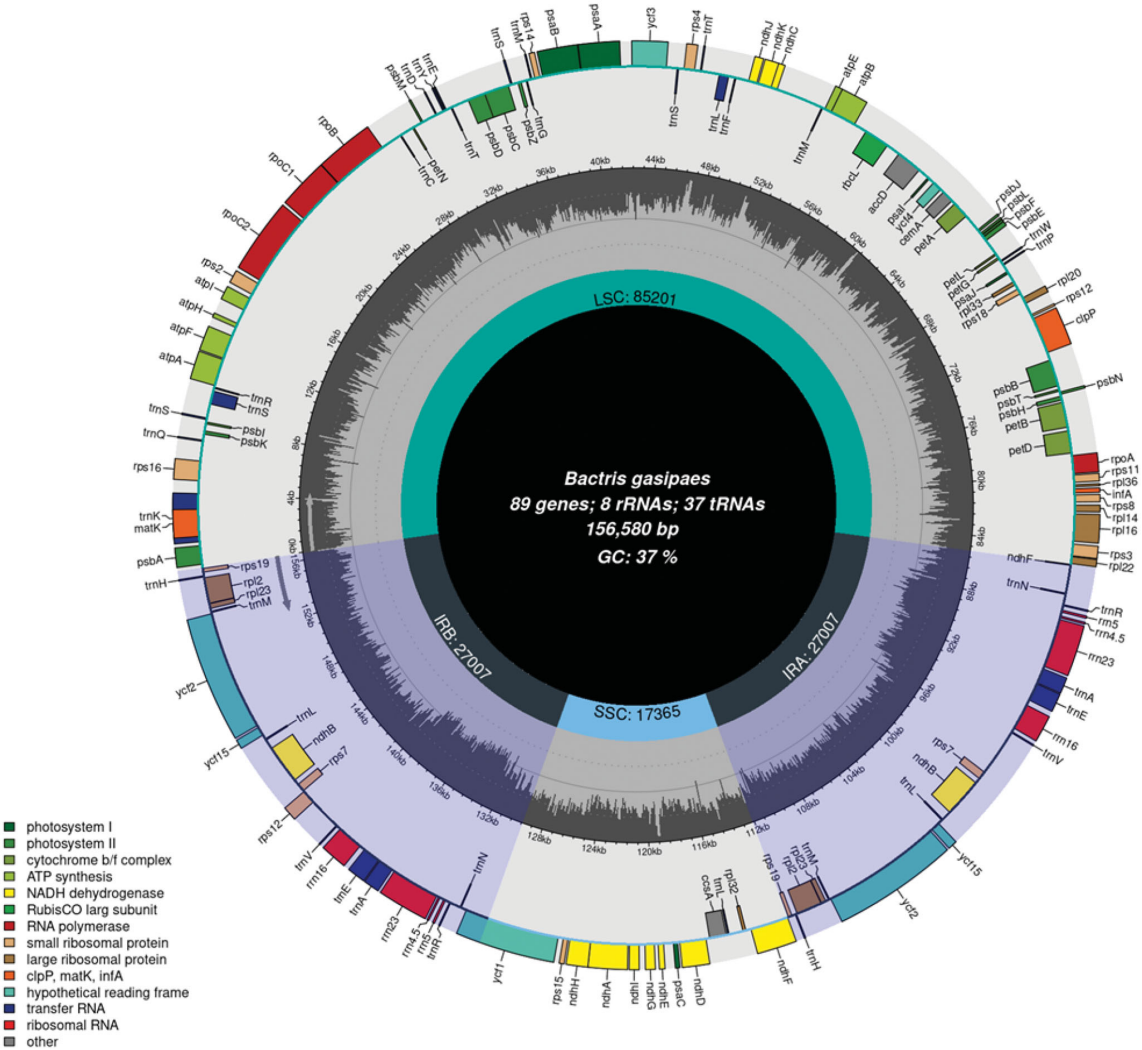
Annotation of the *B. gasipaes* Kunth var. *gasipaes* chloroplast genome. Information including the number of genes, rRNAs and tRNAs and %GC is presented in the inner circle. Large single copy (LSC), Single short copy (SSC) and inverted repeat regions (IRA and IRB) are marked. The outer circle represents the genes annotated, classified by color based on their function.

Among the 10 million pair end reads, 174,750 reads were retained for assembly, giving an average depth of coverage of 190 X. The size of the reconstructed chloroplast genome of *B. gasipaes* Kunth var. *gasipaes* was 156,580 bp (Figure 1). A comparison between the *B. gasipaes* Kunth var. *gasipaes* and *B. gasipaes* Kunth var. *chichagui* genomes showed a contiguity through all the sequence and the presence of two inverted repetitions, common to the majority of plant chloroplast genomes (Supplementary Figure 1; Heinhorst and Cannon 1993). Even though the chloroplast sequences between these two varieties are highly similar, we observed 20 SNVs (Single Nucleotide Variants), 17 insertions of 1 base, 3 insertions of 2 bases, 2 insertions of 3 bases, 1 insertion of 4 bases and 2 insertions of 6 bases in the *B. gasipaes* Kunth var. *gasipaes* sequence. Also, we observed seven regions with mismatches, including a region with 20 unidentified nucleotides in the *B. gasipaes* Kunth var. *chichagui* that was resolved in the *B. gasipaes* Kunth var. *gasipaes* sequence assembly. We identified ten of these mutations in different coding sequences including *matK, rpoB* and *psaA* gene, among others (Supplementary table 1). CPGAVAS2 has identified 61 microsatellites for the *B. gasipaes* Kunth var. *chichagui* and 68 for *B. gasipaes* Kunth var. *gasipaes.* Among those, four are specific to the var. *chichagui* plastome and nine are specific to the var. *gasipaes.* Eighteen are conserved between both plastome, but with a length variation (Supplementary table 2).

Based on this sequence comparison between the *B. gasipaes* Kunth var. *chichagui* and *B. gasipaes* Kunth var. *gasipaes* plastomes, discrimination between these two accessions can be considered at the molecular level. DNA barcoding relies on coding and non-coding plastid markers to identify species. Generally, it is recommended to use the two plastids *rbcL*+*matk* coding regions (core markers) with eventually additional markers (CBOL Plant Working Group 2009) such as *trnH-psbA*, *atpF-atpH*, *psbK-psbI* or *trnL* (Hollingsworth et al. 2011). For this species, two barcodes *rbcL* (NCBI accessions JQ590428, JQ590427, JQ590426) and *matK* (JQ586697, JQ586696, JQ586695) have been developed by the International Barcode of Life project (iBOL; http://ibol.org). Of these, only *matk* shows a variation at one base and therefore could potentially be used to discriminate between wild and cultivated *B. gasipaes*. However, our sampling size is minimal and more samples should be sequenced to confirm this. A study using several accessions of both wild and cultivated *B. gasipaes* individuals did not find any variation for two non-coding plastid markers *trnD-trnT* and *trnQ-rps16* (Couvreur et al. 2007). Alternatively, full plastomes could be used as ultra-barcodes to distinguished more reliably wild and cultivated accessions as was done in Cacao (Kane et al. 2012). Finally, different predicted chloroplast microsatellite markers could also be used for this purpose but would still need to be tested and validated.

Moreover, we annotated 89 genes, 37 tRNAs and 8 rRNAs. When comparing junctions between inverted repeats and single copy regions, we observed differences in distance between genes and junctions compared with *B. gasipaes* Kunth var. *chichagui* and other related species. These positions are crucial to understand chloroplast genome evolution because they are related with chloroplast sequence expansion or contraction (Amiryousefi et al. 2018).

**Figure 2.**
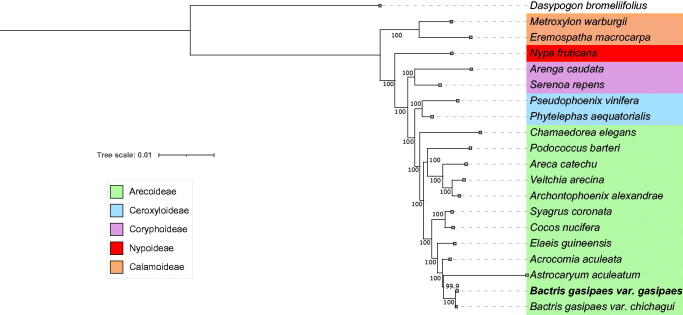
Phylogenetic tree reconstructed with complete chloroplast genome sequences, using MAFFT to align and RAxML to construct the tree, bootstrap values are shown in the branches and species names from the same subfamilies are represented in different colors (***B. gasipaes* Kunth var. *gasipaes*** OM047178*, B. gasipaes* Kunth var. *chichagui* NC_058634.1 (Santos da Silva et al. 2021)*, Acrocomia aculeata* NC_037084.1, *Astrocaryum aculeatum* NC_044482.1 (de Santana Lopes et al. 2018), *Cocos nucifera* NC_022417.1 (Huang et al. 2013), *Syagrus coronata* NC_029241.1, *Elaeis guineensis* NC_017602.1 (Uthaipaisanwong et al. 2012), *Archontophoenix alexandrae* NC_046017.1 (Liu et al. 2020), *Areca catechu* NC_050163.1 (Rajesh et al. 2020), *Veitchia arecina* NC_029950.1, *Chamaedorea elegans* NC_051509.1, *Podococcus barteri* NC_027276.1 (Bethune et al. 2019), *Phytelephas aequatorialis* NC_029957.1 (Barrett et al. 2016), *Pseudophoenix vinifera* NC_020364.1 (Barrett et al. 2013), *Arenga caudata* NC_029971.1 (Barrett et al. 2016), *Serenoa repens* NC_029953.1 (Barrett et al. 2016), *Nypa fruticans* NC_029958.1 (Barrett et al. 2016), *Eremospatha macrocarpa* NC_029964.1 (Barrett et al. 2016), *Metroxylon warburgii* NC_029959.1 (Barrett et al. 2016), *Dasypogon bromeliifolius* NC_020367.1 (Barrett et al. 2013).

The phylogenetic analysis was based on 138,382 aligned sites with no gaps and we identified previously described relationships (Figure 2), congruent with previous studies with the family (Baker et al. 2009). Indeed, phylogenetic relations between subfamilies were well supported (bootstrap support > 94). Both varieties of *B. gasipaes* were recovered with maximum support as sister varieties within the Bactridineae tribe as found in previous phylogenetic studies of short plastid markers (Couvreur et al. 2007).

This resource will be useful for unraveling the domestication history of the cultivated variety (Clement et al. 2017), in particular from the perspective of seed dispersal.

## Supplementary Material

Supplemental Material

## Data Availability

The genome sequence data that support the findings of this study are openly available in Zenodo (doi: 10.5281/zenodo.6337604) and GenBank of NCBI under the accession number: OM047178. The associated Bioproject, Bio-Sample and SRA, are PRJNA825158, SAMN27503645, and SRR18697342, respectively.
