# Supplementary material for: Bactris gasipaes Kunth var. gasipaes complete plastome and phylogenetic analysis
Source: Mitochondrial DNA B Resour. 2022 Aug 26;7(8):1540–4. doi: 10.1080/23802359.2022.2109437 (PMC9423826; doi:10.1080/23802359.2022.2109437)
Supplement: Supplemental Material [file TMDN_A_2109437_SM4133.docx]

***Bactris gasipaes* Kunth var. *gasipaes* complete chloroplast genome and phylogenetic analysis**

Maria Camila Buitrago ^1*^, Rommel Montúfar ^4,+^, Romain Guyot ^2,3^, Cedric Mariac ^2^, Timothy J. Tranbarger ^2^, Silvia Restrepo ^1^, Thomas L.P. Couvreur ^2,4,+^

**Affiliations**

1 Laboratorio de Micología y Fitopatología, Universidad de los Andes, Bogotá, Colombia.

2 DIADE, Univ Montpellier, CIRAD, IRD, Montpellier, France.

3 Department of Electronics and Automation Universidad Autónoma de Manizales, Manizales, Colombia

4 Facultad de Ciencias Exactas y Naturales, Pontificia Universidad Católica del Ecuador, Av. 12 de Octubre 1076 y Vicente Ramón Roca, Quito. rjmontufar@puce.edu.ec

*Current address: 612 Wilson Road, 140 Plant Biology Laboratory, East Lansing, MI 48824-1312.

Corresponding authors: Rommel Montúfar, Thomas Couvreur

- These authors should be considered as co-senior authors


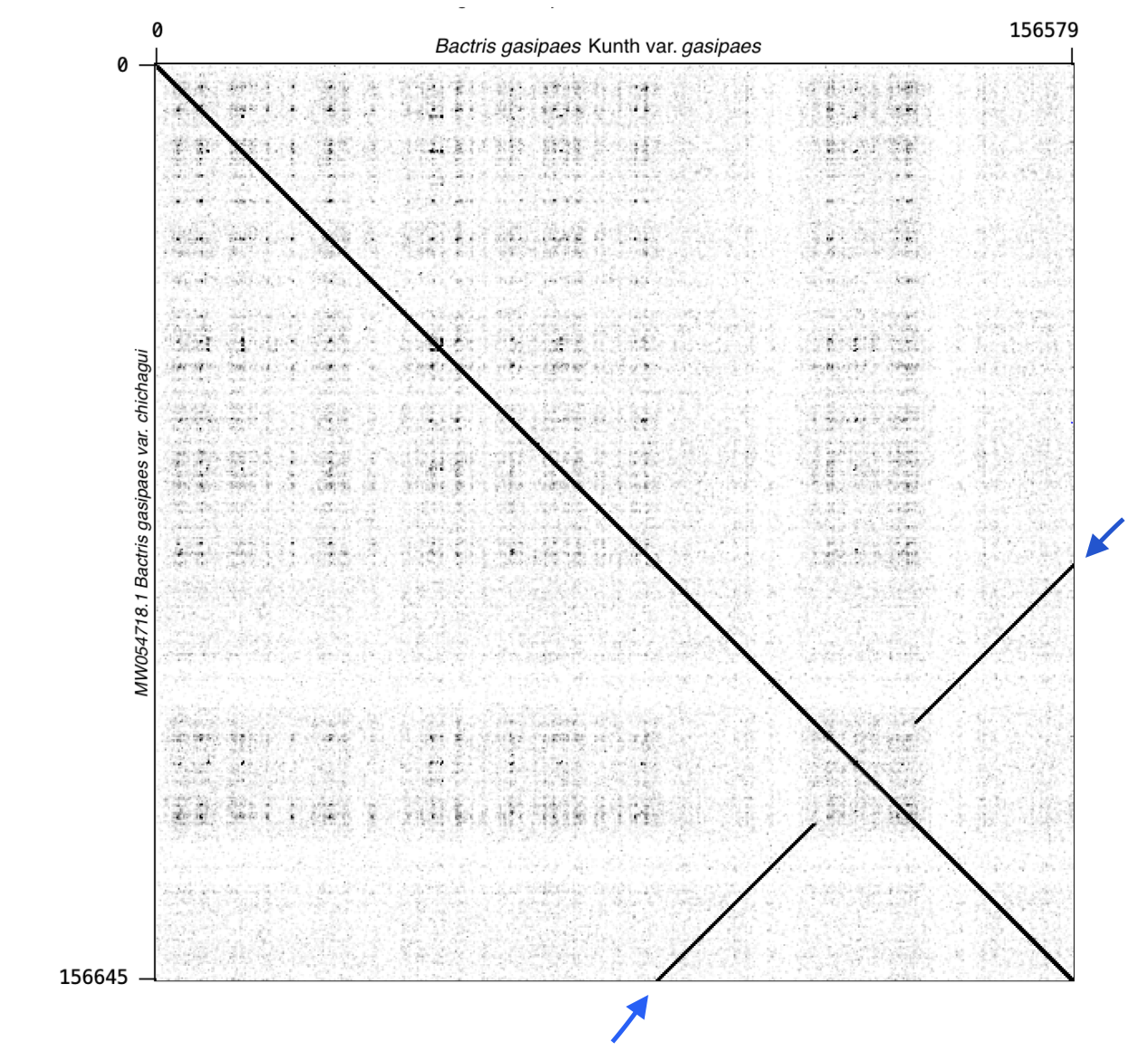
**Supplementary Figure 1**. Dot-plot of *B. gasipaes* Kunth var. *gasipaes* (OM047178) and *B. gasipaes* Kunth var. *chichagui* (NC_058634.1) chloroplast genome sequences. Inverted repetitions are indicated by blue arrows.


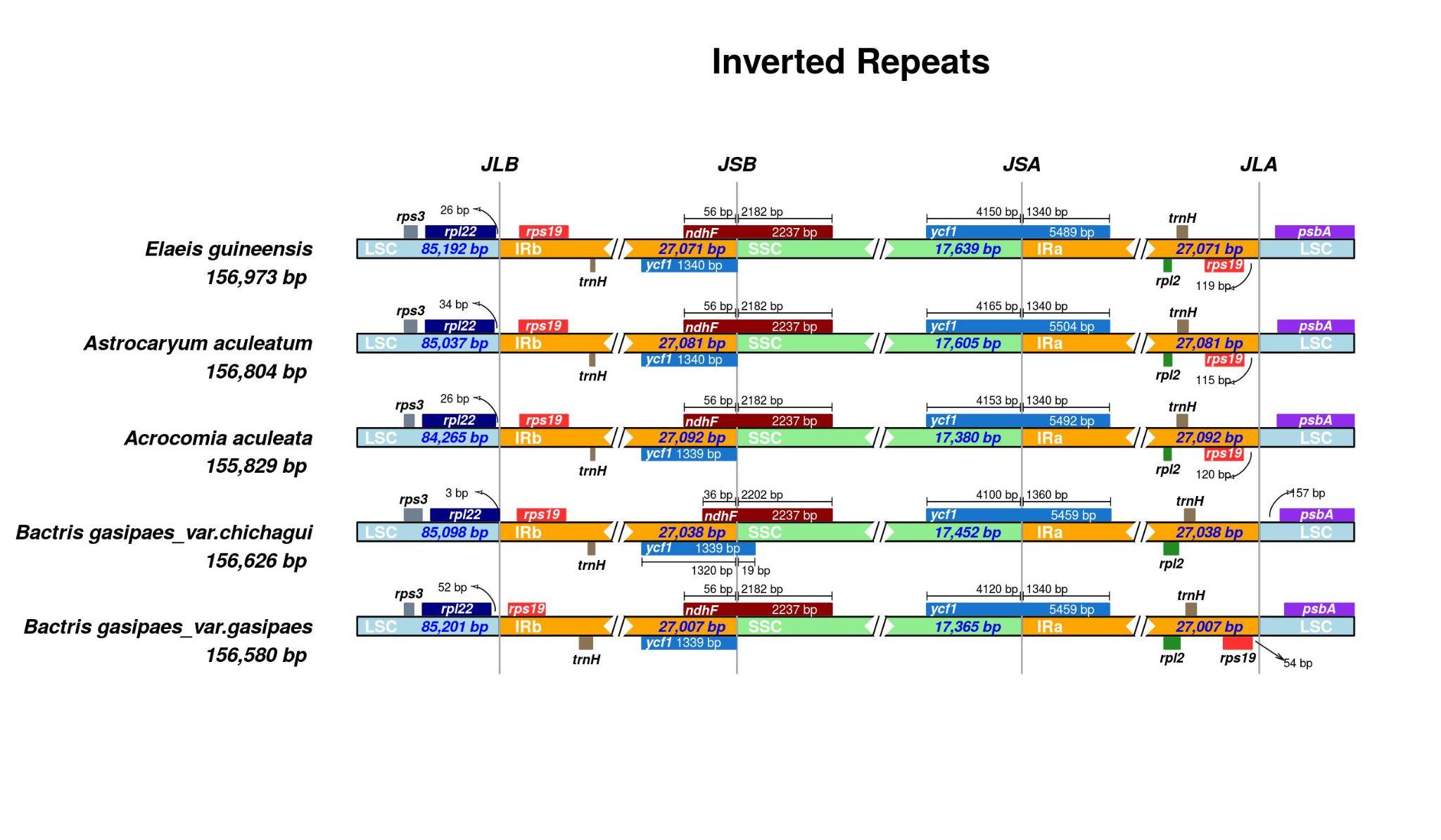


**Supplementary Figure 2.** Visualization of genes present in junctions of LSC-IRb (JLB), IRb-SSC (LSB), SSC-IRa (JSA) and IRa-LSC (JLA). Five chloroplast genomes are depicted: *B. gasipaes* Kunth var. *gasipaes* OM047178*, B. gasipaes* Kunth var. *chichagui* NC_058634.1*, Acrocomia aculeata* NC_037084.1, *Astrocaryum aculeatum* NC_044482.1, *Elaeis guineensis* NC_017602.1. LSC: Large single copy , SSC: IRA and IRB: Single short copy and inverted repeat regions.

**Supplementary table 1.** Mutations between *B. gasipaes* Kunth var. *gasipaes* OM047178*, B. gasipaes* Kunth var. *chichagui* NC_058634.1 plastomes found in coding sequences. SNV: single nucleotide variant.

| **Type** | **Nucleotide**  *B. gasipaes* Kunth var. *gasipaes* | **Nucleotide**  *B. gasipaes* Kunth var. *chichagui* | **Gene** | **Position**  *B. gasipaes* Kunth var. *gasipaes* | **Position**  *B. gasipaes* Kunth var. *chichagui* |
| --- | --- | --- | --- | --- | --- |
| SNV | A | C | *matK* | 2568 | 2602 |
| SNV | G | T | *rpoB* | 24900 | 24932 |
| SNV | C | T | *rpoB* | 25799 | 25843 |
| SNV | G | A | *psaA* | 40081 | 40109 |
| Indel | A | - | *cemA* | 61970 | 61989 |
| SNV | T | G | *rps3* | 84440 | 84453 |
| SNV | A | G | *ndhF* | 112295 | 112310 |
| SNV | T | C | *ccsA* | 116168 | 116168 |
| Substitution | AACAC | GTGTT | *ycf1* | 126539 | 126684 |
| SNV | A | G | *ycf1* | 129383 | 129485 |

**Supplementary table 2.** Micro-satellites analysis with CPGAVAS2 showing variations between *B. gasipaes* Kunth var. *gasipaes* OM047178*, B. gasipaes* Kunth var. *chichagui* NC_058634.1 plastomes.

| *NC_058634.1 Bactris gasipaes var. chichagui plastome* | | | | | | ***OM047178 Bactris gasipaes_var. gasipaes plastome*** | | | | | |
| --- | --- | --- | --- | --- | --- | --- | --- | --- | --- | --- | --- |
| SSR nr. | SSR type | SSR | size | start | end | SSR nr. | SSR type | SSR | size | start | end |
| 1 | p1 | (A)13 | 13 | 3635 | 3647 | 1 | p1 | (A)14 | 14 | 3600 | 3613 |
| 3 | p1 | (C)12 | 12 | 4871 | 4882 | 3 | p1 | (C)13 | 13 | 4831 | 4843 |
| 6 | c | (T)13attttggagagttcgaaaaaaaaagaaagatttttgacttgtctttttttttcccttat(A)13 | 85 | 7446 | 7530 | 6 | c | (T)16attttggagagttcgaaaaaaaaagaaagatttttgacttgtctttttttttcccttat(A)12 | 87 | 7407 | 7493 |
| 8 | p1 | (T)13 | 13 | 13486 | 13498 | 8 | p1 | (T)11 | 11 | 13455 | 13465 |
| 15 | p1 | (T)15 | 15 | 23035 | 23049 | 15 | p1 | (T)16 | 16 | 23003 | 23018 |
| 16 | p1 | (T)10 | 10 | 23354 | 23363 |  |  |  |  |  |  |
|  |  |  |  |  |  | 16 | p1 | (A)10 | 10 | 28372 | 28381 |
|  |  |  |  |  |  | 17 | p1 | (A)10 | 10 | 29032 | 29041 |
|  |  |  |  |  |  | 19 | p1 | (A)10 | 10 | 29613 | 29622 |
|  |  |  |  |  |  | 20 | p1 | (T)10 | 10 | 30226 | 30235 |
| 22 | p1 | (A)11 | 11 | 42911 | 42921 | 25 | p1 | (A)10 | 10 | 42883 | 42892 |
| 23 | p1 | (A)13 | 13 | 47063 | 47075 | 26 | p1 | (A)14 | 14 | 47055 | 47068 |
| 25 | p2 | (AT)12 | 24 | 48601 | 48624 | 28 | p2 | (AT)14 | 28 | 48594 | 48621 |
|  |  |  |  |  |  | 29 | p1 | (A)11 | 11 | 51183 | 51193 |
| 28 | p1 | (T)10 | 10 | 53630 | 53639 | 32 | p1 | (T)11 | 11 | 53629 | 53639 |
|  |  |  |  |  |  | 33 | c | (C)10(A)10 | 20 | 58437 | 58456 |
| 30 | p1 | (A)10 | 10 | 61968 | 61977 | 35 | p1 | (A)11 | 11 | 61970 | 61980 |
| 32 | p1 | (A)12 | 12 | 67679 | 67690 | 37 | p1 | (A)13 | 13 | 67682 | 67694 |
| 33 | p2 | (AT)6 | 12 | 68793 | 68804 | 38 | p2 | (AT)7 | 14 | 68797 | 68810 |
| 35 | p1 | (T)10 | 10 | 71421 | 71430 |  |  |  |  |  |  |
| 36 | p1 | (T)10 | 10 | 71690 | 71699 | 40 | p1 | (T)11 | 11 | 71695 | 71705 |
| 40 | p1 | (T)14 | 14 | 81639 | 81652 | 44 | p1 | (T)15 | 15 | 81645 | 81659 |
| 41 | p1 | (T)11 | 11 | 83737 | 83747 | 45 | p1 | (T)10 | 10 | 83378 | 83387 |
| 42 | p1 | (T)10 | 10 | 85127 | 85136 |  |  |  |  |  |  |
|  |  |  |  |  |  | 46 | p1 | (T)11 | 11 | 83745 | 83755 |
| 44 | p1 | (A)10 | 10 | 114496 | 114505 |  |  |  |  |  |  |
|  |  |  |  |  |  | 48 | c | (A)10tgtaatattaatatatatattatttgttattttatgttaaatgttaaattatgttaaatgttgaaagt(A)10 | 88 | 114425 | 114512 |
| 45 | p1 | (T)10 | 10 | 115035 | 115044 | 49 | p1 | (T)11 | 11 | 115048 | 115058 |
| 46 | p1 | (A)11 | 11 | 115232 | 115242 | 50 | p1 | (A)10 | 10 | 115246 | 115255 |
|  |  |  |  |  |  | 51 | p1 | (T)10 | 10 | 116895 | 116904 |
| 54 | p1 | (A)10 | 10 | 156587 | 156596 | 59 | p1 | (A)12 | 12 | 156506 | 156517 |
